# “For-Profit Philanthropy: Elite Power and the Threat of Limited Liability Companies, Donor-Advised Funds, and Strategic Corporate Giving” By Dana Brakman Reiser and Steven A. Dean. Published in 2022 by Oxford University Press, 329 pages. Reviewed by Giedre Lideikyte Huber

**DOI:** 10.1515/npf-2023-0025

**Published:** 2023-04-04

**Authors:** Giedre Lideikyte Huber

**Affiliations:** Senior Lecturer at the Faculty of Law, University of Geneva, Geneva Center for Philanthropy, Geneve 1211, Switzerland

What is for-profit philanthropy and why should we care? The oxymoron in the title immediately arouses interest, however it is all but a reading bait: Brakman Reiser and Dean swiftly convince us that the subject is highly important and deserves a deep and critical analysis.

The book explores fundamental systemic transformations of the vast US philanthropic sector that occurred essentially during the last two decades. The main message conveyed by the authors is that the lines between business and charity are blurring or even disappearing. Based on multiple case studies, they show that the US regulatory system, which was meant to keep those two areas apart is being eroded. Indeed, practices, players and standards that were originally conceived for the commercial world create a new phenomenon designated as “for-profit philanthropy”. Its scale, the authors argue, has been understudied, and it deserves a particular attention because for-profit philanthropy is rapidly taking over traditional forms of high-profile philanthropy, such as foundations. The benefits but also the risks of such a systemic change merit the attention of policy makers: a major threat is the eradication of public trust, which is the essential objective of the charity law.

The book develops those arguments in three parts.

“Elite philanthropy in the twenty-first century” outlines the legal trends in the contemporary US philanthropy. It focuses, as indicated already in the subtitle, on three types of high-profile charity trends, which the authors consider as the core of for-profit philanthropy: Limited Liability Companies (LLCs), Donor-Advised Funds, and Strategic Corporate Giving. Each category is discussed separately, highlighting advantages and risks.

The authors show that LLCs – a very popular commercial legal vehicle – is increasingly establishing itself in the high-end philanthropic sector, at the cost of traditional foundation structures. Its most prominent examples are philanthropic LLCs of notorious tech entrepreneurs – such as the Chan Zuckerberg Initiative; however, the use of LLCs for philanthropic activities is not limited to this particular generation, and in fact started in more traditional industries, such as oil. The reason why LLCs attract elite philanthropists is arguably their immense flexibility. The restrictions on investments, spending, management practices and publishing of accounts, to which ordinary US foundations are subjected, do not apply. It is true that they do not provide tax advantages for philanthropists that come with “classic” foundations; yet, the authors highlight that the ultra-high net worth US individuals are largely unconcerned by income taxation and thus related tax benefits no longer incentivize them (the tax framework, including marginal income tax rates, has changed significantly in the last 50 years). Flexibility and extreme privacy – or, in other words, the opacity of philanthropic activities appears as a very attractive alternative in this context.

Donor-advised funds (DAFs) also offer greater flexibility than classic foundations, and even provide tax advantages for donors. Affiliated to commercial financial institutions, DAFs accept and hold donations earmarked for future distribution to charities. However, upon the transfer to DAFs, donors do not need to identify the distribution date and/or receiving charity and retain the right to advise the fund on distributions – this right can even be passed on to heirs. Notwithstanding the fact that money may “sleep” for years in DAFs, donors receive immediate “charitable” tax deductions. Brakman Reiser and Dean show that this type of arrangement does not only provide flexibility, but also greater privacy: donor advised funds are less transparent than classical foundations, as they merely publish aggregated data, without identification of individual donors and their grantmaking decisions.

Finally, strategic corporate philanthropy is also singled out as potentially problematic. The authors argue that in its contemporary state, commercial and non-profit goals are often impossible to disentangle. Providing a rare perspective on the development of corporate philanthropy (the first corporations were *only* charitable and religious organisations!), case studies show different rationales justifying corporate giving, also analyzing its links with Corporate social responsibility (CSR). Large multinationals increasingly align charitable giving with their business models, and there is a trend of dismantling separate corporate foundations and transferring their grant-making activities to specific departments within the sponsoring company. Corporate giving is used for a wide variety of purposes that are very distant from classic charity, including the development of new product and markets, smoothing supply chains and attracting as well as retaining talent. Although these activities might produce a “kinder capitalism”, its price, the authors argue, must not be forgotten. Here again, donors emerge as winners at the expense of broader societal interests and the regulatory framework is no longer able to ensure that corporate “giving” truly serves the public instead of business objectives.

The second part, named “Protecting the partnership between elites and the public”, provides a thought-provoking interpretation of the rationale behind the historical development of the US charity laws. Brakman Reiser and Dean argue that this legal framework, enacted in the late 1960s and concerning mainly foundations, essentially sought to improve public trust in elite philanthropy. The Grand Bargain, as they name it, did it through targeting, timing and transparency requirements: 1) targeting requirements limited purposes to which philanthropic funds may be directed, excluding self-dealing and political contributions; 2) timing requirements ensured that philanthropic assets are put to use and prevented exclusively hoarding them; and 3) transparency requirements (e.g. publishing the foundation’s annual statements) brought elite charity into the open so that the public and media could examine its claims of serving the public good. In return, the legislator offered tax advantages, a rather wide power for donors to decide what a public good is and ultimately, a reputational protection. Those rules, not unlike many charity laws outside the US, established a modest but essential public control and, according to the authors, successfully increased public trust, resulting in a “golden age” of philanthropy in the US.

Alas, the for-profit philanthropy rejects targeting, timing, transparency requirements that form the core of charity law, and this has tipped the scales too far in favor of unrestricted liberty for the wealthiest donors. Brakman Reiser and Dean are not proponents of the old order – they acknowledge both that the Grand Bargain rules have aged, and that for-profit philanthropy offers potential to unlock innovation and attract new capital. The authors nonetheless voice their concern regarding the declining public trust in this process, which seems to escape the legislator’s attention.

Consequently, the third part of this book, “Restoring public voice”, aims at providing paths towards rebuilding public trust in philanthropy. The range of responses – similarly to risks – is wide. Brakman Reiser and Dean start with the most realistic proposals, which would be tailored governmental interventions aiming at the current abuses of targeting, timing, and transparency requirements for each of three forms of for-profit philanthropy (ex. requiring DAFs to publicly name the recipients of dormant assets). Such “small” victories, that would consider the needs of the contemporary philanthropy, would already, authors claim, solidify public trust. In addition, carefully designed self-regulatory commitments by philanthropists themselves could have some impact in aligning the public’s concerns about the use of their generosity. However, the most efficient but also ambitious solution remains a radical legislative reform – a More Perfect Bargain. It would, Brakman Reiser and Dean suggest, inject modernized values into the current system and restore philanthropic balance targeting the wealthiest Americans.

Such targeting could be carried out through higher tax rates, more potent policing and even an entirely new form of taxation – for instance, the introduction of a wealth tax and linking charitable tax concessions to it. To the reviewer’s knowledge, such solutions are rare across the world, as wealth tax only exists in a few countries – France has had this legislative experience with incentivizing charitable giving and it did prove efficient,1Literature on this subject is not abundant, but I would like to cite Cagé, Julia, and Malka Guillot. “Is Charitable Giving Political? Evidence from Wealth and Income Tax Returns.” Evidence from Wealth and Income Tax Returns (June 30, 2021) (2021). Those authors exploit the 2017 wealth-tax reform in France, when a change in the taxable base that led to a drop of two third in the number of liable households and, as a result, an increase in the price of charitable giving and changes in giving behavior. therefore this solution appears particularly interesting. Another original suggestion, completely unrelated to taxes, would be to address the elite power directly – for example, enacting corporate law provisions that enable the inclusion of workers and other stakeholders in corporate governance. Such a democratic institutional design could divert corporate philanthropy to serve interests beyond those of shareholders.

Overall, one of the principal strengths of this book lies in this conceptualization of the emergence of for-profit philanthropy and opening of the discussion about the responsibility of law in brokering public trust in situations that verge on the limits of the democracy. Public policy justifications behind charity laws are often hazy, tax and other concessions given to donors being presented as mainly aimed to incentivize charitable giving rather than building trust. Reframing the debate more inclusively like Brakman Reiser and Dean do, i.e. considering not only the contract between the legislator and donors but also between the public and donors seems meaningful and might be more effective in finding new solutions. The authors clearly lay out why public trust in philanthropy is important and give instructions on how to proceed in re-negotiating it.

We might regret that the book addresses nearly exclusively elite philanthropy. The authors discuss DAFs and their use by smaller-scale donors as well as the fact that DAFs democratized giving, but this debate remains accessory. Nonetheless, this choice comes out as a pragmatic one if we consider the characteristics of the US philanthropy market, which is heavily reliant on elite giving – research shows that the share of donations accounted for by a minority of top donors rose sharply over 1960–2012.2Duquette, Nicolas J. “The evolving distribution of giving in the United States.” Nonprofit and Voluntary Sector Quarterly 50.5 (2021): 1102–1116.

Beyond the content, the book is beautifully written and rich both with legal and cultural references, which is particularly heartening to this reviewer who is accustomed to (much) more pragmatic tax law texts. Consider this phrase, an original and acute definition of philanthropy (and I have seen many!): “*Philanthropy relies on an improbable collaboration, with the market’s victors teaming up with its victims to gamble on their shared future*”. Sharp and elegant, the book succeeds in effortlessly drawing the reader into stimulating contemplations and is highly rewarding.

